# Editorial Correspondence

**Published:** 1877-11

**Authors:** Frank A. Ramsy

**Affiliations:** Knoxville, Tenn.


### EDITORIAL CORRESPONDENCE.

KNOXVILLE, TENN., Nov. 11, 1877.

*Editor Atlanta Medical and Surgical Journal:*

In the October number of your JOURNAL you say, "Fractures in utero are to us novel." This is doubtless true with all of us. Nevertheless such an occurrence presented itself to my observation many years ago.

In the days of slavery my family cook, a mulatto, had for a husband a free negro, to whom I gave employment. On a Sunday he and his wife were returning, on horseback, from a country ride, and when within a square of my house the horse she was riding had a fright and threw her. The ground was soft from recent rain, hence her fall did her no injury, except that her left leg fell apon a hay pole, which had been cast off by an outgoing wagon. There was not even the mark of a bruise on the leg. She was about seven months pregnant. About dark of the next day she was in labor, and in a few hours was delivered. The child was alive, but soon died. On the surface of the left leg, extending diagonally near the middle of the leg, and corresponding to the portions of the mother's leg that had been in contact with the hay-pole when she fell, was a bruised line, well marked, distinctly so. And both bones of the child's leg were broken. The mother of the child is now, I believe, in Chattanooga.

The *British and Foreign Medical Review*, Oct., 1840, quoting from another journal, refers to fractures of cranium and upper extremities as not unusual, as occurring during parturition; but I presume that the cases of your former and present correspondent do not belong to that category.

Respectfully, DR. FRANK A. RAMSY.

